# Supplementary material for: A novel diagnostic approach for assessing pulmonary blood flow distribution using conventional X-ray angiography
Source: PLoS One. 2021 Jun 29;16(6):e0253565. doi: 10.1371/journal.pone.0253565 (PMC8241113; doi:10.1371/journal.pone.0253565)
Supplement: Attachment — Submitted filename: 3_ResponseToReviewer_20210422.docx [file pone.0253565.s002.docx]

Response to Reviewers

We wish to thank the editors and reviewers for the constructive suggestions in relation to the submission of the original article on “A novel diagnostic approach for pulmonary blood flow distribution assessment using conventional X-ray angiography” (PONE-D-20-19770). We have revised the manuscript in accordance with your suggestions, and revisions are indicated in red in a file labeled ‘Revised Manuscript with Track Changes’.

In this revised manuscript, we increased the number of patients to demonstrate the clinical relevance of our new method of measuring pulmonary blood flow distribution in each lung using X-ray pulmonary angiography.

PONE-D-20-19770

A novel diagnostic approach for assessing pulmonary blood flow distribution using conventional X-ray angiography

PLOS ONE

Journal Requirements:

Please review your reference list to ensure that it is complete and correct. If you have cited papers that have been retracted, please include the rationale for doing so in the manuscript text, or remove these references and replace them with relevant current references. Any changes to the reference list should be mentioned in the rebuttal letter that accompanies your revised manuscript. If you need to cite a retracted article, indicate the article’s retracted status in the References list and also include a citation and full reference for the retraction notice.

When submitting your revision, we need you to address these additional requirements.

1. Please ensure that your manuscript meets PLOS ONE's style requirements, including those for file naming. The PLOS ONE style templates can be found at

https://journals.plos.org/plosone/s/file?id=wjVg/PLOSOne_formatting_sample_main_body.pdf and

https://journals.plos.org/plosone/s/file?id=ba62/PLOSOne_formatting_sample_title_authors_affiliations.pdf

REPLY: We have amended manuscript to meet PLOS ONE’s style requirements.

2. We suggest you thoroughly copyedit your manuscript for language usage, spelling, and grammar. If you do not know anyone who can help you do this, you may wish to consider employing a professional scientific editing service.

Whilst you may use any professional scientific editing service of your choice, PLOS has partnered with both American Journal Experts (AJE) and Editage to provide discounted services to PLOS authors. Both organizations have experience helping authors meet PLOS guidelines and can provide language editing, translation, manuscript formatting, and figure formatting to ensure your manuscript meets our submission guidelines. To take advantage of our partnership with AJE, visit the AJE website (http://learn.aje.com/plos/) for a 15% discount off AJE services. To take advantage of our partnership with Editage, visit the Editage website (www.editage.com) and enter referral code PLOSEDIT for a 15% discount off Editage services. If the PLOS editorial team finds any language issues in text that either AJE or Editage has edited, the service provider will re-edit the text for free.

Upon resubmission, please provide the following:

The name of the colleague or the details of the professional service that edited your manuscript

A copy of your manuscript showing your changes by either highlighting them or using track changes (uploaded as a *supporting information* file)

A clean copy of the edited manuscript (uploaded as the new *manuscript* file)

REPLY: Our manuscript have been copyedited by our North American colleague named Dr. Hatem Mehrez with certification, as Editorial office suggested.

3. You indicated that you had ethical approval for your study. In your Methods section, please ensure you have also stated whether you obtained consent from parents or guardians of the minors included in the study or whether the research ethics committee or IRB specifically waived the need for their consent.

REPLY: We had obtained written informed consent from patients’ parents. We have added a statement about obtaining consent in the Material and Methods section.

4. Thank you for including your ethics statement:

"The Institutional Review Board of Nagano Children’s Hospital approved all procedures. (IRI-28-1)".

i) Please amend your current ethics statement to confirm that your named institutional review board or ethics committee specifically approved this study.

ii) Once you have amended this/these statement(s) in the Methods section of the manuscript, please add the same text to the “Ethics Statement” field of the submission form (via “Edit Submission”).

For additional information about PLOS ONE ethical requirements for human subjects research, please refer to http://journals.plos.org/plosone/s/submission-guidelines#loc-human-subjects-research.

REPLY: We have amended ethics statement in the Material and Methods section, an attached the IRB approval with English translation.

5. Thank you for stating the following in the Financial Disclosure section:

"This research was funded by Cannon Medical Systems Corporation."

We note that one or more of the authors have an affiliation to the commercial funders of this research study : Canon Medical Systems Corporation.

5.1. Please provide an amended Funding Statement declaring this commercial affiliation, as well as a statement regarding the Role of Funders in your study. If the funding organization did not play a role in the study design, data collection and analysis, decision to publish, or preparation of the manuscript and only provided financial support in the form of authors' salaries and/or research materials, please review your statements relating to the author contributions, and ensure you have specifically and accurately indicated the role(s) that these authors had in your study. You can update author roles in the Author Contributions section of the online submission form.

Please also include the following statement within your amended Funding Statement.

“The funder provided support in the form of salaries for authors [insert relevant initials], but did not have any additional role in the study design, data collection and analysis, decision to publish, or preparation of the manuscript. The specific roles of these authors are articulated in the ‘author contributions’ section.”

If your commercial affiliation did play a role in your study, please state and explain this role within your updated Funding Statement.

REPLY: We have added Funding section and declare commercial affiliation as the editor showed.

5.2. Please also provide an updated Competing Interests Statement declaring this commercial affiliation along with any other relevant declarations relating to employment, consultancy, patents, products in development, or marketed products, etc.

Within your Competing Interests Statement, please confirm that this commercial affiliation does not alter your adherence to all PLOS ONE policies on sharing data and materials by including the following statement: "This does not alter our adherence to PLOS ONE policies on sharing data and materials.” (as detailed online in our guide for authors http://journals.plos.org/plosone/s/competing-interests). If this adherence statement is not accurate and there are restrictions on sharing of data and/or materials, please state these. Please note that we cannot proceed with consideration of your article until this information has been declared.

REPLY: We have added Competing Interests Statement also in Funding section.

Please include both an updated Funding Statement and Competing Interests Statement in your cover letter. We will change the online submission form on your behalf.

Please know it is PLOS ONE policy for corresponding authors to declare, on behalf of all authors, all potential competing interests for the purposes of transparency. PLOS defines a competing interest as anything that interferes with, or could reasonably be perceived as interfering with, the full and objective presentation, peer review, editorial decision-making, or publication of research or non-research articles submitted to one of the journals. Competing interests can be financial or non-financial, professional, or personal. Competing interests can arise in relationship to an organization or another person. Please follow this link to our website for more details on competing interests: http://journals.plos.org/plosone/s/competing-interests

[Note: HTML markup is below. Please do not edit.]

Reviewers' comments:

Reviewer's Responses to Questions

Comments to the Author

1. Is the manuscript technically sound, and do the data support the conclusions?

The manuscript must describe a technically sound piece of scientific research with data that supports the conclusions. Experiments must have been conducted rigorously, with appropriate controls, replication, and sample sizes. The conclusions must be drawn appropriately based on the data presented.

Reviewer #1: Yes

Reviewer #2: Yes

2. Has the statistical analysis been performed appropriately and rigorously?

Reviewer #1: N/A

Reviewer #2: N/A

3. Have the authors made all data underlying the findings in their manuscript fully available?

The PLOS Data policy requires authors to make all data underlying the findings described in their manuscript fully available without restriction, with rare exception (please refer to the Data Availability Statement in the manuscript PDF file). The data should be provided as part of the manuscript or its supporting information, or deposited to a public repository. For example, in addition to summary statistics, the data points behind means, medians and variance measures should be available. If there are restrictions on publicly sharing data—e.g. participant privacy or use of data from a third party—those must be specified.

Reviewer #1: Yes

Reviewer #2: Yes

4. Is the manuscript presented in an intelligible fashion and written in standard English?

PLOS ONE does not copyedit accepted manuscripts, so the language in submitted articles must be clear, correct, and unambiguous. Any typographical or grammatical errors should be corrected at revision, so please note any specific errors here.

Reviewer #1: Yes

Reviewer #2: Yes

5. Review Comments to the Author

Please use the space provided to explain your answers to the questions above. You may also include additional comments for the author, including concerns about dual publication, research ethics, or publication ethics. (Please upload your review as an attachment if it exceeds 20,000 characters)

Reviewer #1: It is a new method and as stated it it is a preliminary study, to verify the theoretical assumption and calculation. Looking at this study at this viewpoint, it is interesting, but without large comparative study it has no clinical relevance, at the moment.

REPLY: In the first manuscript, we had conducted the study as a pilot study. However, we have increased the number of patients from 3 to 7 to demonstrate clinical relevance and to meet the statistically required sample size in this revised manuscript. The number of the cases to test correlation between angiography and lung scintigraphy was estimated by Fisher Z transformation with setting confidence interval as 0.6 and predicted confidence coefficient as >0.8. This description was added in Material and Methods section.

Reviewer #2: The authors presented an interesting pilot study evaluating the feasibility and accuracy of X-ray pulmonary angiography to measure pulmonary blood flow distribution in each lung compared to lung perfusion scintigraphy. The manuscript is with merit and the findings are worth reporting. However, before publication could be considered, the authors should revise the manuscript and address the following concerns.

- MATERIAL AND METHODS

o Participants: the authors should provide an indication of the criteria used to select the study patients

REPLY: We have added inclusion and exclusion criteria for this study in Material and Methods section, as “ The patients whose pulmonary blood flow was supplied by multiple vessels or who had extra blood supply besides main pulmonary artery, who had a lack imaging of lung field, who had overlapped image of main pulmonary artery, were excluded.”

o Statistics: The author should provide some justification of the study n

REPLY: We have added a sentence to show the statistical justification of the sample size in Material and Methods section, as “The sample size of the patients in this pilot study was estimated by was estimated by Fisher Z transformation with setting confidence interval as 0.6 and predicted confidence coefficient as >0.8.”

- RESULTS

o The authors should indicate in the first paragraph of the results the characteristics of the study participants (demographics, age, gender, diagnosis etc)

REPLY: We have added Table 1 which represents demographic data of the enrolled patients.

Minor revisions

- Line 336: “Our method” should be replaced with “our method” (the letter “o” should not be capitalized after a colon)

REPLY: We have uncapitalized the word “Our”.

- Figures/Tables: please revise all the legends and provide the complete explanation of the abbreviations used.

REPLY: We have added explanations of the abbreviations used in figures and tables.

6. PLOS authors have the option to publish the peer review history of their article (what does this mean?). If published, this will include your full peer review and any attached files.

If you choose “no”, your identity will remain anonymous but your review may still be made public.

Do you want your identity to be public for this peer review? For information about this choice, including consent withdrawal, please see our Privacy Policy.

Reviewer #1: No

Reviewer #2: No
